# The Challenges of Nursing Students in the Clinical Learning Environment: A Qualitative Study

**DOI:** 10.1155/2016/1846178

**Published:** 2016-06-05

**Authors:** Nahid Jamshidi, Zahra Molazem, Farkhondeh Sharif, Camellia Torabizadeh, Majid Najafi Kalyani

**Affiliations:** ^1^Faculty of Nursing & Midwifery, Shiraz University of Medical Sciences, Shiraz 71936 13119, Iran; ^2^School of Nursing, Fasa University of Medical Sciences, Fasa 74616 86688, Iran

## Abstract

*Background/Aim*. Clinical learning is a main part of nursing education. Students' exposure to clinical learning environment is one of the most important factors affecting the teaching-learning process in clinical settings. Identifying challenges of nursing students in the clinical learning environment could improve training and enhance the quality of its planning and promotion of the students. We aimed to explore Iranian nursing students' challenges in the clinical learning environment.* Materials and Methods*. This is a qualitative study using the content analysis approach. The participants consisted of seventeen nursing students and three nursing instructors. The participants were selected through purposive sampling method and attended semistructured interviews and focus groups.* Results*. Three themes emerged after data analysis, including ineffective communications, inadequate readiness, and emotional reactions.* Conclusion*. Nursing students in Iran are faced with many challenges in the clinical learning environment. All challenges identified in this study affected the students' learning in clinical setting. Therefore, we recommend that the instructors prepare students with a specific focus on their communication and psychological needs.

## 1. Introduction 

Nurses' competence is based on the knowledge and skill taught to them [1]. Nursing training is a combination of theoretical and practical learning experiences that enable nursing students to acquire the knowledge, skills, and attitudes for providing nursing care [2]. Nursing education is composed of two complementary parts: theoretical training and practical training [3]. A large part of nursing education is carried out in clinical environments [4]. In Iran and many other countries, clinical education forms more than half of the formal educational courses in nursing [5]. Therefore, clinical education is considered to be an essential and integral part of the nursing education program [6]. Since nursing is a performance-based profession, clinical learning environments play an important role in the acquisition of professional abilities and train the nursing students to enter the nursing profession and become a registered nurse [7]. Moreover, the clinical area of nursing education is of great importance for nursing students in the selection or rejection of nursing as a profession [8].

Unlike classroom education, clinical training in nursing occurs in a complex clinical learning environment which is influenced by many factors [9]. This environment provides an opportunity for nursing students to learn experimentally and to convert theoretical knowledge to a variety of mental, psychological, and psychomotor skills which are of significance for patient care [10]. Students' exposure and preparation to enter the clinical setting are one of the important factors affecting the quality of clinical education [11].

Since an optimal clinical learning environment has a positive impact on the students' professional development, a poor learning environment can have adverse effects on their professional development process [8]. The unpredictable nature of the clinical training environment can create some problems for nursing students [12].

The researchers' experience in the nursing clinical education reveals that nursing students' behaviors and performances change in the clinical setting. This change can negatively affect their learning, progress in patient care, and professional performance. Identifying problems and challenges with which these students are faced in the clinical learning environment can help stakeholders solve these problems and contribute to them becoming professional as well as their professional survival [11].

Failure to identify the challenges and problems the students are faced with in the clinical learning environment prevents them from effective learning and growth. As a result, the growth and development of their skills will be influenced [4]. Studies show that the students' noneffective exposure to the clinical learning environment has increased dropout rates. Some nursing students have left the profession as a result of challenges they face in the clinical setting [13].

Many studies have been done on the clinical environment. Some relevant studies have also been carried out in our country; however, most of them have focused on clinical evaluation or stress factors in the clinical training. One study showed that nursing students are vulnerable in the clinical environment and this reduces their satisfaction with the clinical training [14]. Moreover, the nursing students' lack of knowledge and skills in the clinical environment can lead to anxiety [15]. Yazdannik and colleagues found that nursing students suffered from inferiority complex after entering the clinic [16].

According to a review of the literature, few studies have been done on the challenges nursing students are faced with in the clinical learning environment in Iran; these challenges are still unknown. Identifying challenges with which nursing students are faced in the clinical learning environment in all dimensions could improve training and enhance the quality of its planning and the promotion of the students. We aimed to explain the challenges of the nursing students in the clinical learning environment.

## 2. Materials and Methods

### 2.1. Study Design

This paper is a part of a larger grounded theory study. Content analysis was used in this qualitative research so that rich and deep information could be obtained from the phenomenon under study [17]. Since qualitative research emphasizes trust, transparency, verifiability, and flexibility, it is considered a good method to develop insight and interpretation in the field of nursing education [18].

### 2.2. Study Participants

Participants in the study included nursing students and instructors from Shiraz University of Medical Sciences, Shiraz, Iran. Clinical nursing instructors were selected in order to access the information of nursing students who had the experience of working at the patients' bedside.

The population in this study consisted of seventeen nursing students from different academic semesters and three clinical nursing instructors. Various groups of students in terms of age, sex, academic semester, and experience of working at the patients' bedside were used in order to achieve deep and extensive data.

### 2.3. Data Collection

In this study, to better understand the challenges of nursing students in dealing with the clinical setting, individual interviews, group interviews, and observation were used. Individual interviews with nursing students and instructors were carried out face-to-face and in a convenient place based on the willingness of the participants in the School of Nursing, Shiraz University of Medical Sciences. Group interviews with nursing students were also performed to achieve a deeper understanding of this phenomenon. A group interview is a way for people to express their experiences and views with regard to a subject in a group and, instead of a researcher, members of a group are responsible for encouraging each other to talk [19]. The individual and group interviews began by asking the participants a general and open question regarding the description of their encounter with the clinical setting, and then some other questions were asked based on the participants' statements and responses [17].

Moreover, some supplementary questions were utilized based on the participants' comments and opinions (e.g., “would you elaborate more on this?” or “what did you mean by saying …?”) to search and complete information.

All the conducted interviews with the participants were recorded and immediately transcribed verbatim after the end of the interview sessions. Each interview lasted about 40 to 70 minutes and was 55 minutes on average. Interviews were continued with participants until the data was saturated and sampling was ended with data saturation [17].

In addition, the observation method was used to investigate the exposure of students to the clinical setting. The observer recorded the students' activities and conversations for further analysis. The observation method in this study focused on the relationship and the behavior of students, patients, staff, and instructors at the clinical setting. During this phase of the study, field notes and reminders were used to analyze the observations.

### 2.4. Data Analysis

Content analysis was used in this research in order to identify and understand the challenges of nursing students in dealing with the clinical setting. This method of analysis is an interpretive process that focuses on the subject and background and explores the similarities and differences between and within different parts of the text [20]. In this method, the script of the interview was read several times by the researcher to reach an overall understanding. The parts related to the experiences of the participants regarding the challenges of encountering the clinical setting were extracted from the interviews and placed in a separate text. Then, words, sentences, and paragraphs relevant to each other in terms of both content and context were merged and coded. Codes and units of meaning were interpreted in the context of the study and compared in terms of similarities and differences. Finally, abstract subclasses were made based on the semantic line [20]. Rethinking about the codes and the subclasses resulted in the extraction of three main categories.

### 2.5. Trustworthiness of Data

In order to validate the data, manuscripts were reviewed and data coding processes were reconducted by the colleagues and the whole process was peer reviewed by an outside observer. Extracted codes were sent to the participants and approved. In order to obtain the variability criterion, the scripts of a number of interviews, codes, and extracted classes were given to several colleagues who were familiar with the methods of analyzing qualitative research and were not present in the process of conducting this study, and the accuracy of the data coding process was evaluated. Furthermore, allocating sufficient time to collect data and maintaining an objective and impartial view further added to the reliability of the research. In order to obtain generalizability across environments, the results of the research were presented to a number of students who had not participated in the study and they were asked to judge the similarity between the results of the research and their own experiences.

### 2.6. Ethical Issues

The Deputy of Research and Bioethics Committee of Shiraz University of Medical Sciences approved this project prior to the beginning of the study. In the current study, in order to consider ethical principles, the purpose of the study was explained to all the participants and informed consent was obtained for each interview and voice recording. The participants were assured of the confidentiality of the data. In addition, the recorded interviews were kept in a safe place and were only accessible by the researcher.

## 3. Results

The participants consisted of seventeen nursing students and three nursing instructors. The students were in the second, third, and fourth year of study and aged 20–23 years. Moreover, three nursing instructors (two women and one man) participated in this study with an age range of 32 to 38 years and a clinical training experience of 5 to 8 years. After analyzing the interviews with the participants regarding the challenges of nursing students in dealing with the clinical learning environment, three main themes emerged: ineffective communication, inadequate readiness, and emotional reactions.

### 3.1. Ineffective Communications

This main category consisted of two subcategories of improper treatment and discrimination.

#### 3.1.1. Improper Treatment

Students encounter some challenges in dealing with clinical learning environment and in interaction with instructors, patients, and department personnel. Many students stated that they had the most interactions with the instructors and believed that the way an instructor treats a student affects their exposure to clinical learning environment. One student stated the following.

*…to be frank, our instructor did not treat us well. Once, I made a mistake and the instructor reprimanded me right at a patient's bed. Companions of the patient never trusted me again. He had many undue rigors…. *

In addition to the improper treatment of instructors toward the students, some behaviors of nurses are also oppressive to students. One of the students described how improperly the department nurse treated her.

*… One day, I was staying at the nursing station with some of my friends. Suddenly, the department nurse appeared and urged us to get away! …you know you are making the department nurse crowded. Quickly put the card indices back…, said the nurse. *

#### 3.1.2. Discrimination

Discrimination is a subcategory that most students had experienced. They were complaining about a series of discriminatory behaviors they were seeing at the bedside that irritated them. According to what the students claimed, the greatest discrimination in the clinical setting was apparent in behaviors of nurses towards students. One of the students said the following.

*… The head nurse of the department tells us to stand up and leave the nursing station whenever we go there and wants us to let medical students sit on chairs…! *

In addition to behavioral discrimination, some students were also upset and complained about discrimination in the use of educational facilities. One of the students participating in the study said the following.

*… Whenever we need the conference room in the department and ask the nurses of the department to give us the key to that room, they simply reject us and say no! This room is only for residents and medical students to use…, they answer. *

### 3.2. Inadequate Readiness

This category includes three subcategories of inadequate knowledge, deficient practical skills, and insufficiently developed communication skills.

#### 3.2.1. Inadequate Knowledge

Many students did not have sufficient knowledge to care at the bedside when dealing with clinical learning environment and providing care to the patients was challenging for them. One of the students said the following.

*… I wanted to give my patient a Pantazol injection; however, I did not know what kind of medication it was. The patient's companion asked what that medication was and to what medication category it belonged. I did not know to what medication category it belonged and did not even know it was used to treat gastric issues. The patient's companion asked me whether it was an antibiotic and I answered I think so…*

#### 3.2.2. Deficient Practical Skills

Clinical environment is a suitable context for learning skills needed to care for patients. However, some of them are considered basic health care skills and any deficit in them affects the quality of care. In this regard, students had difficulties in performing procedures in some situations, due to the lack of necessary skills. One of the participants said the following.

*Our professor urged us to care for a patient. However, I did not know how to take his blood pressure. The reason was that I could not recognize the sound….*

Deficiency in practical skills in caring for patients was a concern of many students in the clinical setting. One of the students stated the following.

*… The first time I took the blood pressure machine and intended to take a patient's blood pressure, I had the blood pressure cuff upside down around his elbow…it was really my fault. I got embarrassed in front of the patient…. *

#### 3.2.3. Insufficiently Developed Communication Skills

Many students mentioned the lack of communication skills as the reason for deficiency in communicating with the clinical learning environment. One of the students participating in the study said the following.

*… Once we wanted to visit a patient who had a shunt along with our professor. There was a bulge on his hand. I asked him what that bulge was in front of the patient. The patient became upset and I myself regretted. The professor told me that I shouldn't have asked that question in front of the patient and he could explain it to me…. *

Insufficiently developed communication skills sometimes cause disruption in providing care for patients. One student participant said the following.

*… the nurse said that she wanted to take blood and urged us to go and watch. She pricked the patient several times because she was unable to find the vein. I got tormented! The patient was screaming out of pain. I told the nurse that she should act gently toward the patient. She got angry and told me not to interfere and not to talk like that in front of the patient and to control ourselves…. *

### 3.3. Emotional Reactions

This class includes the following two subcategories of stress and inferiority complex.

#### 3.3.1. Stress

Many of the students participating in this study became distressed and overwhelmed in dealing with new experiences within the clinical learning environment. From the perspective of these students, providing care for patients is stressful to them. One of the students said the following.

*… Once I wanted to examine a patient, I was so stressful…I was afraid of doing something that causes harm to the patient. I was giving his medication with a great fear and was praying for him to stay safe. I was hoping for my internship to be just finished as soon as possible….*

The presence in the clinical setting and exposure to new events cause emotional reactions in students. Such reactions have a significant effect on their learning process. One of the students participating in the study said the following.

*… When I went to the department, I saw a patient to whom some devices were attached. I was full of stress, as I had never seen such a situation. My hands and legs were shaking. I could not concentrate at all….*

#### 3.3.2. Inferiority Complex

Inferiority complex was more evident among female students than male ones. In this regard, one of the students said the following.

*…once I wanted to place an IV cannula inside an old man's vein who was hospitalized. I am not a lab rat, the patient shouted. Since then, I feel I have lost my self-confidence every time I want to insert an IV cannula…I have that old man's image in my mind all the time, and I'm worried of making another mess…. *

Students in lower semesters experienced greater inferiority than students in higher semesters. In this regard, one of the instructors said the following.

*… Students often do not have enough confidence early in their internship…they gradually become more confident as they get used to the hospital and its environment…. *

## 4. Discussion

The findings obtained from the study demonstrated that ineffective communication, inadequate preparation, and emotional reactions are Iranian nursing students' challenges in the clinical learning environment.

It is one of the teachers' major responsibilities to treat nursing students properly in the clinic, causing higher enthusiasm and motivation for learning as well as increasing their self-confidence [4]. Nabolsi et al. [2] demonstrated in their study that proper treatment and establishment of a communication with students are an important item for nursing teachers to be a role model for students. Training that involves value and respect facilitates the teaching-learning process and socializes the students into the nursing profession [2]. The results of the studies conducted by Baltimore and Sharif and Masoumi demonstrate that conflicts and improper treatment between the staff and students negatively affect the clinical teaching trend [15, 21]. Hanifi and colleagues found that proper communication with students increased their motivation [22].

Many of the students participating in the study complained about the staff's discrimination between them and students of medicine. The result of the study conducted by Mohebbi and coworkers in Iran demonstrated that a high percentage of nursing students reported discrimination between them and students of other fields [23]. In Baraz-Pordanjani et al.'s study, discrimination in the use of educational facilities and amenities and also in interpersonal communication was reported as a factor distorting the nursing students' professional identity in the clinic [24], which is in line with the results of our study. The comparison between nursing and medicine and regarding medicine as a superior major violates nursing students' personal dignity and gives them a sense of professional inferiority [25]. Students' inadequate preparation for entering the clinical environment creates problems for them and nursing teachers [26]. Even though they learn the fundamentals of nursing in classrooms and practice rooms, nursing students do not have sufficient time to practice and repeat these skills to completely enter the clinic. Killam and Heerschap found that the students' insufficient practice and lack of skill before entering the clinical environment created problems for them with respect to learning in the clinic [27]. Moreover, the students' lack of skill in confronting the clinical environment and dealing with actual patients is evident [11]. Students' lack of knowledge and skill and inadequate preparation for entering the clinical environment disturb their learning processes and make them anxious [28]. Acquisition of communication skills in nursing students creates a guiding atmosphere in the clinical environment, followed by an increase in their motivation [22]. Nursing students' lack of practical skills is considered as a challenge in entering the clinical environment [29].

Nursing students' stress in confronting the clinical environment affects their general health and disturbs their learning processes [30]. According to one study, stress is one of these students' experiences in the clinical environment [11]. In Changiz et al.'s (2012) study, it was revealed that the causes of nursing students' stress in the clinical environment fall into three types of stress due to the educational plan, stress due to the educational environment, and factors concerning the students [31]. In Chesser-Smyth's (2005) study, stress and anxiety were one of the students' experiences in the clinical environment [8]. Nursing students' young age when entering the clinical environment and their social and emotional lack of experience lead to stress and psychological problems [32].

An inferiority complex is another challenge mentioned by the students participating in the study. The results of Edwards et al.'s [30] study showed that low self-confidence is one of the nursing students' problems. Adequate self-confidence is one of the nursing students' requirements in providing good care [33]. In Joolaee et al.'s (2015) study, lack of self-confidence has been referred to as a major cause of fear and anxiety in nursing students. The researcher demonstrated in his study that lack of self-confidence also disturbs communication in nursing students [11]. Moreover, having adequate self-confidence for caregiving is one of the most important factors affecting the students' learning [34]. In Begley and White's (2003) study, self-confidence was an important part of a nurse's personal and professional identity [35]. We found that nursing students in Iran are faced with many challenges in the clinical learning environment, which affect their professionalization and learning processes. Many students are not mentally prepared to enter the clinical environment leading to higher rates of psychological problems. Moreover, lack of adequate knowledge and skill along with lack of mental and psychological preparation disturbs the learning and patient caregiving processes. Improper treatment, discrimination, inadequate knowledge and skill, and lack of communication skills in these patients lead to stress and inferiority complexes in them. In view of the students' challenges in confrontation with the clinical learning environment and the necessity of learning and providing patients with care in a peaceful environment free of any tension, educational authorities and nursing faculties are required to pay particular attention to these issues and try to facilitate the nursing students' learning and professionalization. Hence, the following can be concluded:

1. Based on the results of the study, many students lack the communication skills necessary for effective communication in the clinical environment. It is suggested that the effective communication skills are taught to students before they enter the clinical environment with the emphasis on the differences between the clinical environment and the classroom environment.
2. In view of the results of the study, many students mentioned lack of theoretical knowledge and practical skills as one of the problems involved in caregiving. Therefore, before students enter the clinical environment, it should be ascertained that they are theoretically and practically prepared as they take tests and give care in the skill lab.
3. In light of the presence of stress and inferiority complexes in students in confronting the clinical environment, it is suggested that while they receive psychological consultation on the nursing profession, caregiving, and the hospital environment plans be made for them to visit the hospital and to get acquainted with the clinical learning environment before they begin the actual internship.

The innovation of this study was that we studied how the nursing students were faced with the clinical learning environment and all components of this process by a grounded theory study (this paper is a part of a larger grounded theory study). In addition, in this study, the challenges of nursing students were deeply assessed with respect to educational, behavioral, emotional, and practical aspects, which differentiates this study from other previous studies.
